# Development of Immediate Release Tablets Containing Calcium Lactate Synthetized from Black Sea Mussel Shells

**DOI:** 10.3390/md20010045

**Published:** 2022-01-02

**Authors:** Magdalena Mititelu, Elena Moroșan, Anca Cecilia Nicoară, Ana Andreea Secăreanu, Adina Magdalena Musuc, Irina Atkinson, Jeanina Pandele Cusu, George Mihai Nițulescu, Emma Adriana Ozon, Iulian Sarbu, Teodora Dalila Balaci

**Affiliations:** 1 Department of Clinical Laboratory and Food Safety, Faculty of Pharmacy, Carol Davila University of Medicine and Pharmacy, 6 Traian Vuia Street, 020945 Bucharest, Romania; magdalena.mititelu@umfcd.ro (M.M.); elena.morosan@umfcd.ro (E.M.); 2 Department of Pharmaceutical Technology and Biopharmacy, Faculty of Pharmacy, Carol Davila University of Medicine and Pharmacy, 6 Traian Vuia Street, 020945 Bucharest, Romania; anca.nicoara@umfcd.ro (A.C.N.); ana.stanescu@umfcd.ro (A.A.S.); teodora.balaci@umfcd.ro (T.D.B.); 3 “Ilie Murgulescu” Institute of Physical Chemistry, 202 Spl. Independentei, 060021 Bucharest, Romania; iatkinson@icf.ro (I.A.); jeanina@icf.ro (J.P.C.); 4 Department of Pharmaceutical Chemistry, Faculty of Pharmacy, Carol Davila University of Medicine and Pharmacy, 6 Traian Vuia Street, 020945 Bucharest, Romania; 5 Department of Pharmaceutical Physics and Biophysics, Drug Industry and Pharmaceutical Biotechnologies, Faculty of Pharmacy, “Titu Maiorescu” University, 004051 Bucharest, Romania

**Keywords:** calcium lactate, mussel shells, physical-chemical characterization, preformulation studies

## Abstract

Nowadays, the use of marine by-products as precursor materials has gained great interest in the extraction and production of chemical compounds with suitable properties and possible pharmaceutical applications. The present paper presents the development of a new immediate release tablet containing calcium lactate obtained from Black Sea mussel shells. Compared with other calcium salts, calcium lactate has good solubility and bioavailability. In the pharmaceutical preparations, calcium lactate was extensively utilized as a calcium source for preventing and treating calcium deficiencies. The physical and chemical characteristics of synthesized calcium lactate were evaluated using Fourier Transform Infrared Spectroscopy, X-ray diffraction analysis and thermal analysis. Further, the various pharmacotechnical properties of the calcium lactate obtained from mussel shells were determined in comparison with an industrial used direct compressible Calcium lactate DC (PURACAL^®^). The obtained results suggest that mussel shell by-products are suitable for the development of chemical compounds with potential applications in the pharmaceutical domain.

## 1. Introduction

The huge growing demand for renewable feedstocks makes marine byproducts one of the most attractive research areas. The transformation of marine-sourced biomass into chemical compounds has attracted attention for the development of renewable compounds. Considering the enormous amount of water sources (around 70% from the planet’s surface), the aquaculture industries become a valuable source of biochemical compounds [1,2,3,4,5,6]. Mussels, a high protein food source, were produced using around 95% from aquaculture methods in the total world mollusk production in 2013 [1]. The exoskeletons of mollusks, such as snails, mussels, oysters are composed mostly (over 90%) of calcium carbonate mixed with a small amount of protein material that gives the carcass strength. Mollusks are of general importance in food chains and as members of ecosystems. Certain species are of direct or indirect commercial and even medical importance to humans. Many mollusks are a source of food for many crops, and therefore, play an important role in the fishing industries of many countries, especially in Asian countries, such as China and Japan. Many species of shellfish are also used to make ornaments and are harvested for the pearl and mother-of-pearl industries [7,8,9].

World aquaculture production (fish, seafood and aquatic plants) reached a historical record in 2018 of 114.5 million tons of live mass. The main producing countries are China, India, Indonesia, Vietnam, Bangladesh, Egypt, Norway and Chile. In recent years, bivalve culture has expanded considerably, China is the largest consumer and producer of mollusks, accounting for about 84% of the global volume grown in 2017. Byproducts resulting from mollusk culture can be used in a variety of industrial products, such as fertilizers, building materials, poultry feed, pharmaceuticals and nutraceuticals [10,11].

One of the possibilities of capitalizing on the waste resulting from the food recovery of mollusks consists in capitalizing on the calcium in the shells. Until now, there are few studies on the cleaning process of the mussel shells in order to obtain calcium carbonate and subsequently, calcium lactate [12,13,14].

Calcium is among the most important mineral from the human body (an adult organism contains 1200 g of calcium) [15]. From the whole quantity of calcium, 99% is presented in teeth and bones and the rest of 1% from cell sap has an important role in muscle contraction, cellular metabolism, neural transmission, etc. [16]. One of the most important sources of calcium is calcium lactate pentahydrate. Compared with other calcium salts, calcium lactate has great bioavailability and good solubility. Calcium lactate is a non-toxic and water-soluble compound used as calcium supplement [17], anti-microbial [18], anti-carcinogen [19], etc.

**Figure 1:**
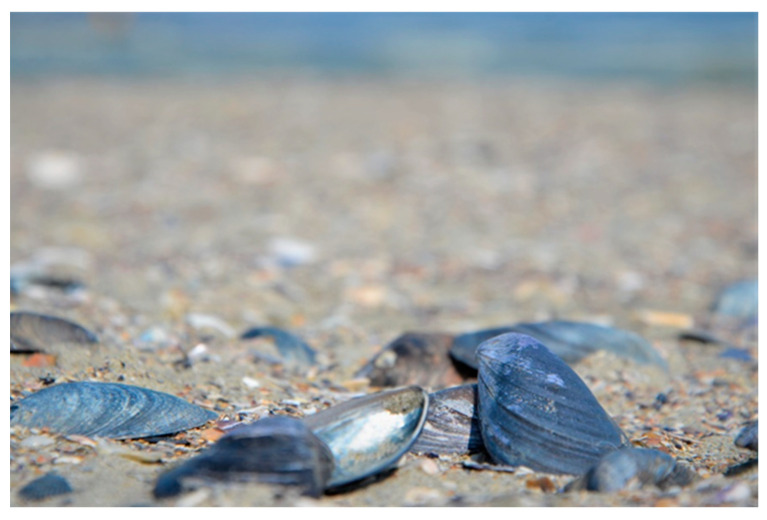
Mussel shells from Romanian Black Sea coast.

The present study developed a way to capitalize on calcium from mussel shells in the form of obtaining calcium lactate for pharmaceutical use. Two species of mussels *Mytilus galloprovincialis* and *Mytilus edulis* are found in abundance in the Black Sea. By processing the mussel shells on the Romanian Black Sea coast (Figure 1), calcium lactate of pharmaceutical purity was obtained [20].

## 2. Results and Discussion

### 2.1. Elemental Analysis

The results of the elemental analysis are presented in Table 1.

**Figure 2:**
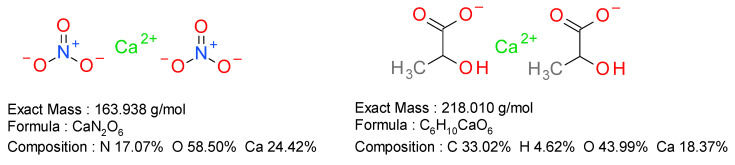
Elemental composition of calcium lactate and calcium nitrate.

Based on the carbon content of the sample of 30.69 ± 0.4% and reported to the expected theoretical content of 33.02%, the content of calcium lactate in the sample was calculated as 92.94 ± 1.21%. The presence of nitrogen in the sample indicates the possibility of the existence of another calcium salt, most probably of calcium nitrate. Figure 2 represents the elemental composition of the two compounds: calcium lactate and calcium nitrate.

Based on the nitrogen content of 1.73 ± 0.4% from the sample and reported to the expected theoretical content of 17.07%, the content of calcium nitrate in the sample was calculated as 10.13 ± 2.34%. The analysis indicates that the sample contains 92.94 ± 1.21% calcium lactate and 10.13 ± 2.34% calcium nitrate. Based on this composition, the average content in calcium is 18.96 ± 0.11%.

According to the elemental quantification, in order to obtain 65 mg calcium ion concentration per tablet, 342.1052 mg of calcium lactate must be included in one dose.

### 2.2. Fourier-Transform Infrared Spectroscopy (FTIR)

**Figure 3:**
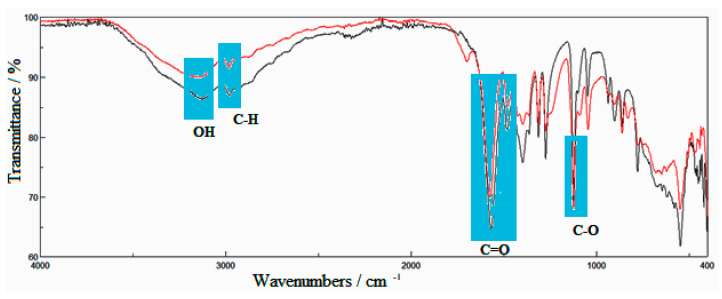
FTIR spectra of synthesized calcium lactate (red line) and standard calcium lactate (black line).

FTIR spectra of synthesized calcium lactate and standard calcium lactate are shown in Figure 3.

From Figure 3 it was observed that the carboxylate group has a large dipole moment and gives two intense peaks, assigned as the asymmetric stretch band registered at 1573.6 cm^−1^ and 1478.2 cm^−1^. The large band at 3157.9 cm^−1^ is produced by the hydroxyl group, while the strong band observed at 1123.3 cm^−1^ is produced by the C-O bond stretching. The band at 2982.4 cm^−1^ is assigned to the C-H bond stretch [21,22].

The FTIR spectra of both types of calcium lactate, synthesized and standard, are very similar as can be observed in Figure 3. The Pearson correlation between the two sets of spectral data is 0.953. The small band observed at 1698.0 cm^−1^ is produced by the small content of calcium nitrate from the composition of the newly synthesized calcium lactate. This band is not visible in the spectrum of the standard calcium lactate.

### 2.3. X-ray Diffraction

**Figure 4:**
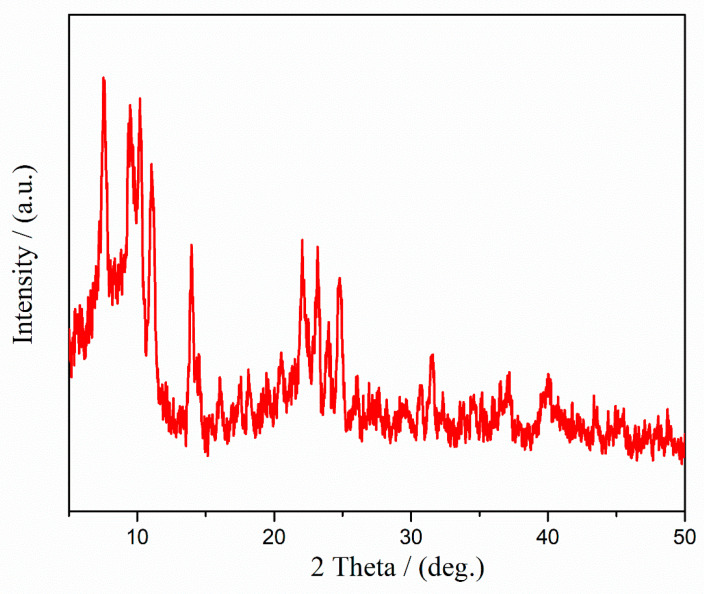
XRD spectrum of synthesized calcium lactate.

The X-ray diffraction (XRD) pattern of synthesized calcium lactate is represented in Figure 4. In Table 2 the peak angles, d-values, and heights for calcium lactate pentahydrate are given according to the reference card PDF card number 00-029-1596 for calcium lactate pentahydrate [23]. The XRD patterns refinement using the Whole Pattern Powder Fitting (WPPF) method confirmed the presence of only the calcium lactate as a single crystalline phase. No other calcium phases were presented. The XRD analysis does not reveal the presence of calcium nitrate may be due to its small quantity in the prepared sample.

### 2.4. Thermal Analysis

**Figure 5:**
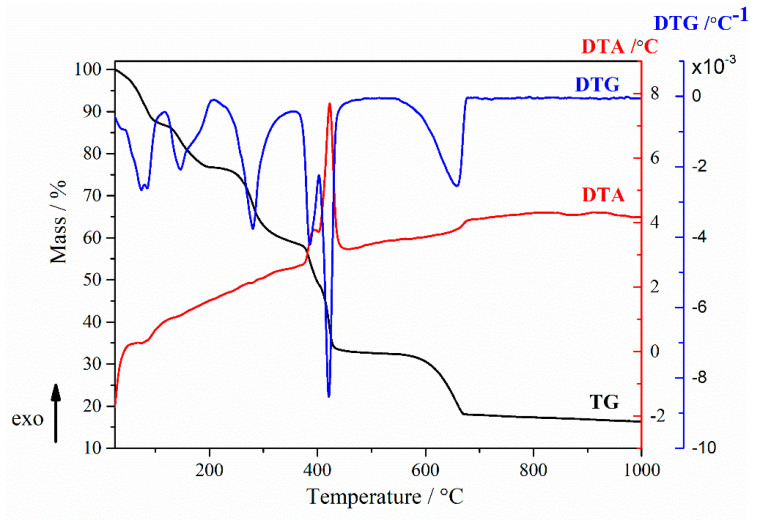
TG/DTA curves of synthesized calcium lactate.

The thermal curves (TGA-DTA-DTG) of the synthesized calcium lactate are shown in Figure 5. As reported in the literature, four mass loss steps are included in the thermal decomposition of calcium lactate pentahydrate [24,25]. The first weight loss step is in two stages: one between 30 and 120 °C (*T*_DTA_ = 51.58 °C/78.83 °C, *T*_DTG_ = 72.75 °C/82.66 °C) and another one between 120 and 200 °C attributed to water molecules from calcium lactate. The TG data agree with the XRD pattern which demonstrates the presence of calcium lactate pentahydrate. The total weight loss for the first step in the temperature range 30–200 °C (*T*_DTG_ = 147.16 °C) corresponds to 23.29%. The second weight loss which appears in the temperature range between 200–373 °C (*T*_DTG_ = 281.25 °C) corresponds to 18.71% of mass loss. The third step between 372–533 °C temperature range (*T*_DTA_ = 393 °C/422.7 °C, *T*_DTG_ = 385.83 °C/420.83 °C) corresponds to a 25.66% weight loss. These two steps are associated with the formation of calcium carbonate [24,25]. The final step between 533–865 °C (*T*_DTA_ = 680 °C, *T*_DTG_ = 657.1 °C) temperature range corresponds to a 15.4% weight loss. The final residue is 17.04% and is represented by calcium oxide. Calcium oxide was formed in a process known as calcination where calcium carbonate loses carbon dioxide through thermal decomposition [25].

### 2.5. Calcium Lactate Pharmacotechnical Properties

Before including a new ingredient in the tablet’s formulation, the pharmacotechnical properties must be determined to establish a correct formulation and to choose the suitable manufacturing method, which leads to a quality final product with satisfactory and predictable characteristics.

The aim is to manufacture the tablets by direct compression technology, this being usually the most desirable method, due to its well-known advantages, especially the lack of moisture needs and the high protection of the active ingredient.

To perform an objective and accurate analysis of the studied calcium lactate characteristics, all pharmacotechnical properties were determined in comparison with an already industrial used direct compressible (DC) sort, Calcium lactate DC (PURACAL^®^) synthetized by PURAC, Netherlands. The pharmacotehnical parameters determined for both tested calcium lactate powders are reported in Table 3.

The calcium lactate synthetized from mussels presented a surprisingly lower content in moisture (8.6%) than the processed DC powder (18.7%), this being a suitable characteristic for the ingredients used in direct compression. 

For both powders, the flowability parameters could not be registered using the 10 mm nozzle, as they were not consistently flowing. When changing with a 15 mm nozzle, they both presented as free-flowing, with no stirring being necessary. In contrast with the results on the loss of drying, the flowability was better for PURACAL^®^, which had a flowing time of 6.44 s, an angle of repose around 26° and the flowing rate was 9.316 g/s. The obtained values are showing that the powder has a good free flow, ideal for manufacturing tablets by direct compression technology. For calcium lactate synthetized from mussels, the flowing time was 25.2 s, with an angle of repose around 32° and a flowing rate of 2.381 g/s, typical behavior for powders with poor free flow. Considering that the flowing was possible without stirring and through a medium diameter nozzle, the results are estimated to be acceptable and must be considered when formulating the tablets. Still, the calcium lactate synthetized from mussels demonstrated a notable flowability in comparison with most of the pharmaceutical ingredients obtained from natural sources. 

The volumetric characteristics also provide useful information and unexpected results. Even the bulk and tapped densities had different values, with a much higher density for the calcium lactate synthetized from mussels, the decreases in volumes after 500 and 1250 tapping were similar, leading to close values for HR and IC. Both materials showed a good flow (1.13 and 1.14 for HR) and very good compressibility (11.93 and 12.38 for IC), but it can be affirmed that the calcium lactate synthetized from mussels displays a better ability for direct compression. The results were not at all predictable, as it is unusual for a powder with a natural origin to have superior compressibility than a material specially processed to fulfill this function [26,27,28,29,30,31].

**Figure 6:**
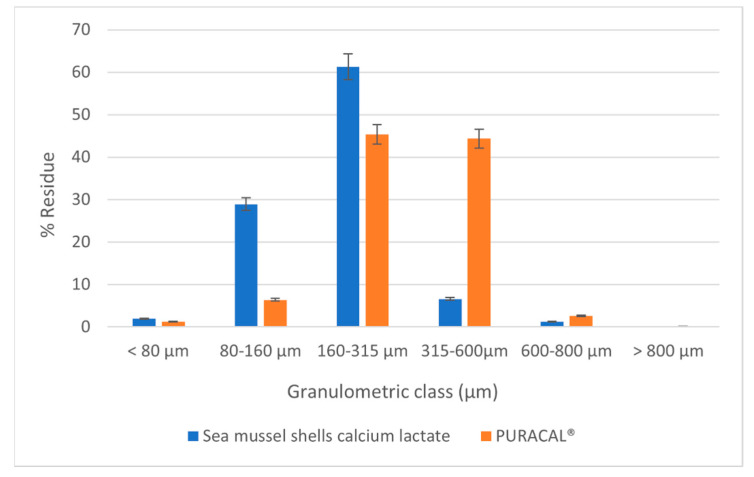
Granulometric analysis of calcium lactate powders.

After the particles dimensions were determined, a histogram (Figure 6) was obtained by representing the distribution of particle size on granulometric classes for both studied formulations.

From Figure 6 it can be noticed that most of the particles belonging to calcium lactate from sea mussels have a particle size between 80 and 315 μm, meantime in the DC calcium lactate a considerable part of the particles have larger dimensions between 160–600 μm. These results well explain the flowability obtained values, as the smaller particles are flowing slowly, and the bigger ones present an excellent flow. Concerning the compressibility, it can be observed exactly the opposite, the particle diameters are justified by the significant difference in the powder’s density, but the preferable compressibility was exhibited by the lower sizes particles.

Considering these aspects determined in the pharmacotechnical powder study, when the formulations for tablets were decided, it must select excipients with very good flowability for the calcium lactate from mussels and with higher compressibility for the DC calcium lactate [32,33].

### 2.6. Tablets Preformulation Studies

Both bulk powders used for direct compression are white, fine and homogenous.

### 2.7. Precompression Studies for Tablets Containing Calcium Lactate

In order to determine the performance of the material during the direct compression and to accurately select the process parameters, different tests were performed on the powder blends. The samples were noted considering the active ingredients: F1—mussels calcium lactate and F2—DC calcium lactate.

The pharmacotechnical characteristics are presented in Table 4.

In terms of moisture content, from Table 4 was observed a drastic decrease of the values for both active ingredients after mixing with the excipients. Humidity low values are ideal for powders processed by direct compression and this beneficial aspect was reached by selecting adequate excipients.

Additionally, a significant improvement in the flowability of both ingredients was noticed. The increase in flow rate is more important for the mussel’s calcium lactate formulation (from 2.381 g/s to 5.714 g/s), as it was initially weaker. If for F1 it was doubled, for F2 the rising was not so remarkable (from 9.316 g/s to 10.563 g/s). PURACAL^®^ showed great flowability anyway, but the main purpose was to associate excipients able to enhance mussels’ calcium lactate flowing performances. The determined results prove that both materials have a good flow behavior suitable for compression technology.

Concerning the compressibility, the changes are not so notable, as the characteristics of raw materials were already good enough for processing into tablets. Still, a slight improvement for both powders was remarked, with a pronounced decrease in the F1 density, advantageous for the tableting as this study was not constrained in using a high compression force [34,35,36].

**Figure 7:**
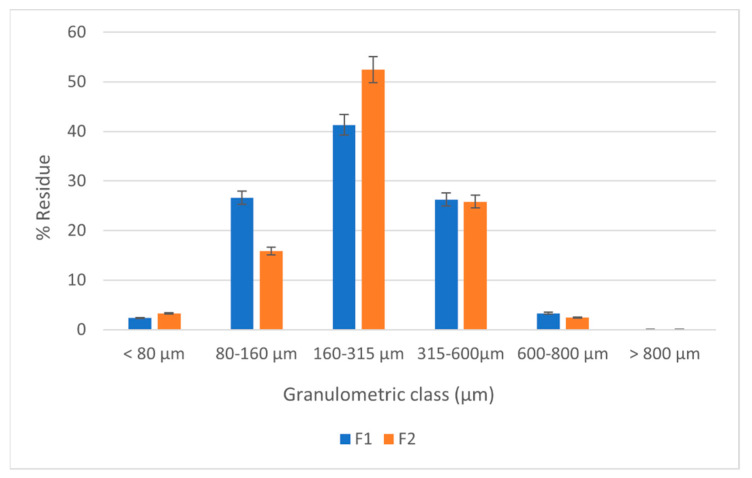
Granulometric analysis of the direct compression materials.

Figure 7 represented the histogram of particle size distribution on granulometric classes for both formulations.

It was also observed that the particle size distribution suffered a relevant change, this directly influenced the flowability of the materials, as the specific test suggested. From Figure 7 a more homogenous distribution on the histogram was observed, with a significant increase of the dimensions, also an opportunity for the compression process.

### 2.8. Quality Parameters of the Tablets

**Figure 8:**
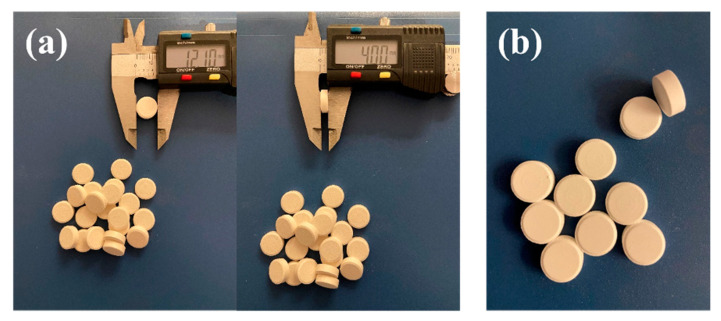
Calcium lactate tablets appearance (**a**) F1 and (**b**) F2.

Round shape uncoated tablets with smooth and uniform appearance, intact edges, a flat surface, white colored, were obtained for both formulations (Figure 8).

The manufactured tablets quality attributes were established by different physical and mechanical characteristics, and the results of the tests are listed in Table 5.

The average sizes of the tablets (thickness 4 mm and diameter 12 mm) are evidence of the uniformity in the structure of the material for direct compression, good flowability and compressibility of the blend, and a well-conducted process. From Table 5 the results for both formulations comply with pharmacopeial criteria and low values for SD show the homogeneity between the tablets of the same batch.

The weight variation for both formulations also conforms to the compendial standard, indicating a consistency of dosage obtained by uniform filling of the mold during the process. The results are typical for formulations with great flow properties.

Concerning the hardness and friability, some differences between formulations were observed. F1 presents a lower mechanical resistance (65.10 ± 4.65 kN) and, also, a lower friability (0.18 ± 0.25%) than F2 (69.30 ± 3.98 kN, respectively 0.31 ± 0.26%). These properties were dependent on the mechanical characteristics of the excipients and on the applied compression force. The desirable hardness was around 60 kN, which ensures a proper disintegration and dissolution of the tablets. The ideal friability is 0% in order to withstand any mechanical shocks. The registered results were indicated that the tablets manufactured according to F1, the formulation containing mussels’ calcium lactate, have better hardness and lower friability [37,38,39,40,41].

The in-vitro disintegration time was around 7 min (418 ± 3 s) for F2 and below 5 min for F1 (288 ± 2 s) proving the significant influence of sodium starch glycolate used as superdisintegrant excipient in the first formulation. Although both formulations are disintegrated in accordance with Pharmacopoeia requirements for immediate release tablets, it was obvious that the formulation containing mussels’ calcium lactate presents a better performance. A quicker release of the active ingredient from the tablets ensures a proper absorption rate and thus it can exert its therapeutic action in the body.

The disintegration time depends on the composition of the tablets, their size and shape and their mechanical strength, which results from the application of compressive force. Disaggregation is considered to be an inverse process to that of compression.

The disintegration process involves solubility of the substance and the nature of the disintegrant, porosity and moisture of the tablet, and the hydrophilicity of the active ingredient [42,43].

F1 presented a higher dissolution rate (96.77 ± 2.85%) than F2 (95.12 ± 3.44%). Both formulations are meeting the imposed Pharmacopoeia limits for immediate release tablets, predictable for high water soluble ingredients, and for systems processed by direct compression. Still, it can be observed the important influence of choosing the right formulation in the development of a new pharmaceutical product and the selection of a superdisintegrant in the composition of tablets [44,45,46,47,48,49]. Our future studies are oriented on developing more tablets formulations containing mussels shell calcium lactate and registering the dissolution profiles of different media, including the digestive fluids simulating ones.

## 3. Materials and Methods

### 3.1. Materials

The raw material used for the calcium lactate synthesis consists of mussels collected from natural populations of the Romanian Black Sea coast.

All used reagents and the reference substances were of analytical purity. They were provided by Fluka^®^ Analytical, Switzerland and Sigma-Aldrich, Germany. The excipients for tablets were purchased from different manufacturers, as follows: Microcrystalline Cellulose 302 from JRS PHARMA GmbH & Co. KG, Rosenberg, Germany, Sodium Starch Glycolate from Maruti Chemicals, Gujarat, India, Maize Starch from HL Agro, Mumbai, India, Magnesium Stearate from Peter Greven, Netherlands and Talc from Peter Greven, Netherlands.

The weighing of the substances was performed with a Mettler Toledo AT261 balance (with 0.01 mg sensitivity).

### 3.2. Synthesis of Calcium Lactate

The mussels were rinsed thoroughly under running water. Flesh and shells were manually separated with a knife. Out of 2.5 kg whole mussels, 0.3 ± 0.05 kg mussel flesh and 1.2 ± 0.2 kg shells were obtained.

The separated shells were carefully rinsed, dried and then ground with a colloidal ball mill, to increase the reactive surface. The powder was deproteinized using a 1% KOH solution to remove the organic components and then, treated with a 30% lactic acid solution.

**Figure 9:**
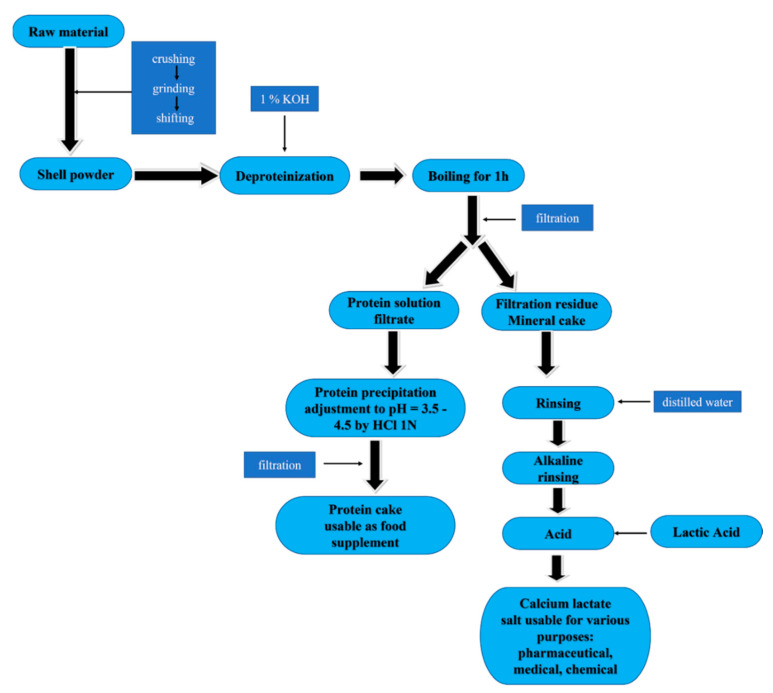
Flowchart for Calcium lactate technological synthesis from mussel shells.

The flowchart of calcium lactate production is shown in Figure 9.

The salt purification is based on the difference in diethyl ether solubility between lactic acid and calcium lactate. The yield calculated on the anhydrous salt in relation to the use of 30% lactic acid was 85.62%.

### 3.3. Elemental Analysis

Quantitative elemental analysis was expressed as a percentage of the quantitative ratios between the atoms of the studied organic compound. A Perkin-Elmer 2400 Series II CHNS/O was used. The samples were weighed in tin microcans using a Perkin Elmer microbalance. The combustion temperature was set at 850° C and in the reduction zone the temperature was 500 °C. Cystine was used as an analytical standard. For each analyzed sample, two determinations were performed, using different amounts from the sample (~1.5 mg and ~2.5 mg). The result was calculated as the average of the two determinations.

### 3.4. Physicochemical Characterization of the Obtained Calcium Lactate

Fourier Transform Infrared spectra (FTIR) were performed in solid phase using a JASCO FT/IR-4200 spectrophotometer equipped with an ATR PRO450-S accessory with diamond optics, working in the attenuated total reflection (ATR) technique in the spectral range of 400–4000 cm^−1^, at a resolution of 4 cm^−1^.

X-ray diffraction (XRD) experiments were performed on powders, using a Rigaku Ultima IV diffractometer in parallel beam geometry equipped with CuKα radiation (wavelength 1.5406 Å). The XRD patterns were collected in the 2θ range between 10 and 70 with a speed of 2°/ min and a step size of 0.02°. PDXL software from Rigaku, connected to the ICDD database was used for phase identification and XRD patterns refinement using the Whole Pattern Powder Fitting (WPPF) method (Rigaku’s PDXL software).

Thermal measurements were performed on a Mettler Toledo TGA/SDTA 851e thermal analyzer apparatus, in an airflow atmosphere with a flow rate of 80 mL min^−1^ and at a heating rate of 10 K min^−1^. The TG curves were recorded from room temperature to 1000 °C. The samples were held in alumina crucibles.

### 3.5. Calcium Lactate Pharmacotechnical Properties

The determined pharmacotehnical characteristics were:

(i) Moisture content, which is important to be reduced in all powders included in solid dosage forms, was assessed as the loss on drying, by the Karl Fisher method using a HR 73 Mettler Toledo halogen humidity analyzer.
(ii) Particle size is highly influencing the behavior of the powder during the manufacturing process, as well as the dissolution profile of the final pharmaceutical form. The sieving and sorting method were applied, using a CISA Sieve Shaker Mod. RP 10, produced by Cisa Cedaceria Industrial, Spain. The powder was passed, under mechanical shaking through a set of sieves with well-known mesh sizes, placed under each other, in ascending order of the finesse degree.
(iii) Flowability, a demanding mechanical characteristic for direct compression technology, was established by the angle of repose, flowing time and rate parameters, registered for 60 g of powder that flows through a standardized diameter nozzle. The study was performed with an Automated Powder and Granulate Testing System PTG-S3, manufactured by Pharma Test Apparatebau GmbH, Germany.
(iv) Compressibility, also a critical property for the manufacturing method, was settled by determining volumetric characteristics (bulk and tapped density) and by calculating the Hausner ratio (HR) and Carr Index (CI), also used for flowability predictions. Vankel Tap Density Tester, produced by Vankel Industries Inc., Palo Alto, CA, USA, was used. First, the bulk density was determined by measuring the volume of 50 g of powder into a graduated cylinder. The tapped volume was measured after applying a different number of mechanical shocks, then the Hausner ratio (HR) was calculated as the ratio between tapped and bulk density. A value under 1.25 was an indication that the powder was free flowing. Carr index is given by the following equation:

(1) $$CI\%=\frac{\left(tapped\;density-bulk\;density\right)\times 100}{tapped\;density}$$

According to literature data, better flowability and compressibility can be obtained if the value of the Carr Index is smaller [50,51,52,53,54].

All tests were performed six times for each powder.

### 3.6. Tablets Preformulation Studies

#### 3.6.1. Excipient Selection

For developing an optimum pharmaceutical form, preformulation studies on the direct compression material must be performed. The final DC powder must have adequate mechanical characteristics. In this context, considering that the active ingredient dosage is 342.105 mg/tablet, the excipients must be carefully selected and efficient in low quantities, as it is not compliant to manufacture high weight tablets. The two active ingredients proved to have different pharmacotechnical characteristics, so it cannot use the same excipients, because they need to meet the individual requirements of the base powder.

For mussels calcium lactate the following were selected: (i) Microcrystalline cellulose 302—a filler excipient with great binding attributes and the best choice to improve active ingredient’s flowability, (ii) Sodium Starch Glycolate, the sodium salt of cross-linked carboxymethyl starch, chosen for its superdisintegrant properties through rapid swelling due to the adsorption of large amounts of water leading to faster disintegration, also having good flow abilities, not characteristic for superdisintegrants excipients, and (iii) magnesium stearate as lubricant.

As excipients for PURACAL^®^, maize starch, a filler and disintegrant excipient suitable for the active ingredients which contain a high amount of moisture and to enhance the blend compressibility were chosen. Its low flowability was the reason for which the two lubricants (magnesium stearate and talc) were added [55,56,57].

#### 3.6.2. Preparation of Direct Compression Powders

The active ingredients (mussels calcium lactate and DC calcium lactate) were mixed with the direct compression excipients in the following ratios: F1—mussels calcium lactate:Microcrystalline cellulose 302:Sodium Starch Glycolate:Magnesium stearate 62.2:35.8:1:1 and F2—PURACAL^®^:Maize Starch:Magnesium stearate:Talc 62.2:34.8:1:2.

The raw materials were accurately weighed.

F1—calcium lactate from mussels, Sodium Starch Glycolate and Microcrystalline cellulose 302 were passed through a 20-mesh sieve, then were mixed and homogenized in V blender for 15 min at 15 rpm, at room temperature. Over blend, the Magnesium Stearate sifted on 40-mesh sieve was added and lubricated for 5 min.

F2—PURACAL^®^ and Maize Starch were similarly mixed for 15 min, then the lubricants were added.

#### 3.6.3. Precompression Studies for Tablets Containing Calcium Lactate

Both obtained direct compression blends were subjected to the same pharmacotechnical studies as the active ingredients, being tested for: moisture content, particle dimensions, flowing and compression abilities, using the same methods already described. All tests were performed six times for each composed powder.

### 3.7. Formulation of the Immediate Release Tablets Containing Calcium Lactate

After determining the characteristics of the powder, the final formulations of the tablets were established as they are listed in Table 6.

### 3.8. Tablets Manufacturing

For the immediate release tablets manufacturing, direct compression technology was selected. A single-post eccentric machine Korsch EK-O type, equipped with 12 mm flat punches, adjusted to the target weight of tablets (550 mg), was used. The applied compression force was low (8–9 kN).

### 3.9. Quality Parameters of the Tablets

After manufacturing, the obtained tablets were tested to determine the quality characteristics of the final products, according to compendial specifications and in force regulations [58,59].

#### 3.9.1. Sizes (Diameter and Thickness)

The uniformity of the tablets’ diameter and thickness depend on the compressed material homogeneity and density or on the right compression force. The dimensions were determined on ten tablets of each formulation with VK 200 Tablet Hardness Tester, produced by Vanderkamp, Cary, NC, USA.

#### 3.9.2. Mass Uniformity

The weight variation is also influenced by the compression parameters. The analysis was done according to European Pharmacopoeia, by individually weighing 30 tablets of each formulation, then calculating the average mass. The tablets are complying with the test if no more than 1 individual mass is outside the limits of 85–115% of the average mass [60].

#### 3.9.3. Mechanical Resistance

The tablets must be sufficiently cohesive to maintain their integrity during handling, packaging, transport and handling. The tablet crushing strength cannot exceed certain limits as it would compromise the disintegrating properties, the release of the active ingredients and even the effectiveness of the product. Tablet hardness was measured on 10 tablets of each formulation, with VK 200 Tablet Hardness Tester. Each tablet was placed on the measuring plate, the mobile part moves at a low speed until the tablet breaks and the load required to crush the tablet is registered.

#### 3.9.4. Friability

Friability is the resistance to abrasion and rolling of a tablet subjected to external forces. The initial weight of 10 tablets was measured and they were placed in the Vankel friabilator, rotating at 25 rpm for 4 min, then weighed again. The difference in the weight was noted and was expressed as a percentage. It should be preferably below 1.0%.

#### 3.9.5. In-Vitro Disintegration Time

A crucial condition for the absorption of the active ingredients from the tablets is their disintegration into primary particles, at a suitable time when they are brought into an aqueous medium. According to European Pharmacopoeia, immediate release tablets must disintegrate in water, at 37 ± 0.5 °C, for a maximum of 15 min. The determination was performed using Erweka DT 3 apparatus, manufactured by Erweka^®^ GmbH, Langen (Hessen), Hessen, Germany. The test was carried out on six tablets of each formulation using distilled water at 37 ± 0.5 °C as a disintegration media. The time taken for complete disintegration of the tablet, with no residue left on the screen, was measured in seconds [60].

#### 3.9.6. In-Vitro Dissolution Rate

The dissolution rate was determined using USP basket Apparatus I (dissolution tester ERWEKA DT 800), in 500 mL of distilled water. The apparatus was settled at 37 ± 0.5 °C, with a rotating speed of 100 rpm and the test was performed on 6 tablets of each formulation. The amount of dissolved calcium lactate was determined after 30 min, by the titrimetric method. After 30 min, 300 mL of each vat were filtered. To 100 mL of filtered solution, 150 mL of water, 2 mL of 3 N hydrochloric acid and 15 mL of 1 N sodium hydroxide were added and stirred. 300 mg of blue hydroxynaphthol were added and titrated with 0.05 M edetate disodium solution to blue [61].

## 4. Conclusions

New manufactured tablets containing Black Sea mussel shell calcium lactate have been formulated. The elemental analysis indicates that the obtained powder contains a higher amount of calcium lactate with small quantities of calcium nitrate. Analysis of physical and chemical properties of synthesized calcium lactate using spectral and thermal analysis methods was performed. The FTIR, XRD and thermal TG-DTG-DTA analyses confirmed the formation of calcium lactate, with no influence of the low nitrogen content. The determined pharmacotechnical properties of the calcium lactate synthesized from mussel shells proved its efficiency for use in the tablet formulation. For assessing the good performance quality of byproduct calcium lactate tablets (F1) a comparison with another already direct compressible calcium lactate PURACAL^®^ (F2) was achieved. The results of the quality test performed on both formulations are proving that in the present study high quality calcium lactate tablets were manufactured. Both formulations pharmacotechnical and in vitro properties were satisfactory and within the limits imposed by rules into force. As the results are similar for F1 and F2, it can be concluded that the physical and mechanical properties of the tablets are not influenced by the type of calcium lactate used. The calcium lactate obtained from Black Sea mussel shells is a strong candidate for the pharmaceutical industry, as it is eco-friendly, not expensive, easy to synthetize, has excellent pharmacotechnical properties and leads to superior quality drug delivery systems.

## Figures and Tables

**Table 1:** Elemental analysis of the obtained calcium lactate.

	C%	H%	N%	S%
Assay values (Synthesized calcium lactate)	30.69 ± 0.40	4.87 ± 0.30	1.73 ± 0.40	0.34 ± 0.05
Theoretical values	33.02	4.62	0.00	0.00

**Table 2:** Tabulated peak angles, d-values, and heights for calcium lactate pentahydrate. In parenthesis are given one standard deviation of the least units cited (PDF card number 00-029-1596).

2 Theta (deg)	d (A)	Height (cps)
7.53 (3)	11.73 (4)	136 (15)
9.49 (9)	9.31 (9)	115 (14)
10.28 (3)	8.60 (2)	137 (15)
11.09 (3)	7.97 (2)	124 (14)
13.98 (4)	6.33 (17)	94 (13)
14.3 (4)	6.17 (6)	27.66 (4)
16.02 (7)	5.53 (2)	25 (6)
17.56 (2)	5.047 (5)	25 (6)
18.22 (7)	4.89 (2)	25 (6)
20.49 (9)	4.31 (7)	14 (5)
22.20 (3)	4.00 (5)	56 (10)
23.20 (4)	3.831 (6)	81 (12)
23.86 (3)	3.726 (5)	51 (9)
24.75 (3)	3.726 (5)	80 (12)
30.74 (9)	2.906 (8)	19 (3)
31.56 (6)	2.832 (5)	38 (8)
37.12 (8)	2.420 (5)	28 (7)
39.99 (7)	2.253 (4)	27 (7)
43.4 (2)	2.082 (10)	10 (4)

**Table 3:** Pharmacotechnical properties for calcium lactate powders.

Parameter	Calcium Lactate Synthetized from Mussels	Calcium Lactate DC (PURACAL^®^)
Moisture content (%)	8.60 ± 0.44	18.70 ± 0.81
Flow time (s) *	25.2 ± 0.79	6.44 ± 0.33
Angle of repose (θ°) *	32.7 ± 0.58	26.22 ± 0.49
Flow rate (g/s) *	2.381	9.316
V_0_ (mL)	59.9 ± 0.18	86.2 ± 0.12
V_500_ (mL)	53 ± 0.23	75.7 ± 0.19
V_1250_ (mL)	52.8 ± 0.08	75.5 ± 0.06
Bulk density (g/mL)	0.834	0.580
Tapped density (g/mL)	0.947	0.662
Hausner’s ratio (HR)	1.13	1.14
Carr Index (CI) (%)	11.93	12.38

* nozzle: 15 mm, no stirring.

**Table 4:** Precompression properties for direct compression powders.

Parameter	F1	F2
Moisture content (%)	5.20 ± 1.28	7.90 ± 1.36
Flow time (s) *	10.5 ± 0.71	5.68 ± 0.58
Angle of repose (θ°) *	24.80 ± 0.61	24.75 ± 0.72
Flow rate (g/s) *	5.714	10.563
V_0_ (ml)	73.4 ± 0.11	82.4 ± 0.18
V_500_ (ml)	65.5 ± 0.19	73.6 ± 0.14
V_1250_ (ml)	64.7 ± 0.24	72.8 ± 0.11
Bulk density (g/mL)	0.681	0.606
Tapped density (g/mL)	0.772	0.686
Hausner’s ratio (HR)	1.13	1.13
Carr Index (CI) (%)	11.78	11.66

* nozzle: 15 mm, no stirring.

**Table 5:** The pharmacotechnical and in vitro evaluation of the calcium lactate tablets.

Tested Parameters	Formulation Code	
	F1	F2
Thickness (mm)	4.00 ± 0.19	4.00 ± 0.24
Diameter (mm)	12.00 ± 0.54	12.00 ± 0.38
Mass uniformity	546.00 ± 2.43	547.00 ± 3.78
Mechanical resistance (N)	65.10 ± 4.65	69.30 ± 3.98
Friability (%)	0.18 ± 0.25	0.31 ± 0.26
In Vitro disintegration time (seconds)	288 ± 2	418 ± 3
In Vitro dissolution rate, after 30 min (%)	96.77 ± 2.85	95.12 ± 3.44

**Table 6:** The formulations for the calcium lactate tablet.

Ingredient	Quantity mg/Tablet		Role in Formulation
	F1	F2	
Calcium lactate from mussel shells	342.10	-	Active ingredient
Calcium lactate DC (PURACAL^®^)	-	342.10	Active ingredient
Microcrystalline cellulose 302	196.90	-	Filler Binder
Sodium Starch Glycolate	5.50	-	Superdisintegrant
Maize Starch	-	191.40	Filler Disintegrant
Magnesium stearate	5.50	5.50	Lubricant
Talc	-	11.00	Lubricant
TOTAL	550	550	
